# Attenuated ability of BACE1 to cleave the amyloid precursor protein via silencing long noncoding RNA *BACE1*-AS expression

**DOI:** 10.3892/mmr.2014.2351

**Published:** 2014-06-23

**Authors:** TE LIU, YONGYI HUANG, JIULIN CHEN, HUIYING CHI, ZHIHUA YU, JIAN WANG, CHUAN CHEN

**Affiliations:** 1 Central Laboratory, Shanghai Geriatric Institute of Chinese Medicine, Longhua Hospital, Shanghai University of Traditional Chinese Medicine, Shanghai 200031, P.R. China; 2 Central Laboratory, School of Life Science and Technology, Tongji University, Shanghai 200092, P.R. China

**Keywords:** β-secretase-1, β-secretase-1-antisense, long noncoding RNAs, noncoding RNAs, amyloid precursor protein

## Abstract

Although large numbers of long noncoding RNAs (lncRNAs) expressed in the mammalian nervous system have been detected, their functions and mechanisms of regulation remain to be fully clarified. It has been reported that the lncRNA antisense transcript for β-secretase-1 (BACE1-AS) is elevated in Alzheimer’s disease (AD) and drives the rapid feed-forward regulation of β-secretase, suggesting that it is critical in AD development. In the present study, the senile plaque (SP) AD SH-SY5Y cell model was established using the synthetic amyloid β-protein (Aβ) 1–42 *in vitro*. Using this model, the potential of siRNA-mediated silencing of lncRNA BACE1-AS expression to attenuate the ability of β-secretase-1 (BACE1) to cleave amyloid precursor protein (APP) and to reduce the production of Aβ_1–42_ oligomers was investigated. MTT assays demonstrated that exogenous Aβ_1–42_ suppressed SH-SY5Y cell proliferation and induced APP-related factor expression and SP formation. Furthermore, quantitative polymerase chain reaction and western blot analysis revealed that the mRNA and protein expression of Aβ_1–42_ and Aβ_1–40_ was significantly increased in the AD model group, with a marked decrease in Ki-67 expression at day six. RNase protection assays (RPA) and northern blotting analysis confirmed that exogenous Aβ_1–42_ not only promoted the expression of the APP-cleaving enzyme BACE1, but also induced lncRNA BACE1-AS expression. Furthermore, lncRNA BACE1-AS formed RNA duplexes and increased the stability of BACE1 mRNA. Downregulation of lncRNA BACE1-AS expression in SH-SY5Y cells by siRNA silencing resulted in the attenuation of the ability of BACE1 to cleave APP and delayed the induction of SP formation in the SP AD SH-SY5Y cell model.

## Introduction

Long noncoding RNAs (lncRNAs), which are a type of noncoding RNA (ncRNA) varying in size from 200 bp to >100 kb, are transcribed by RNA polymerase II, and are often spliced and polyadenylated (1–4). They have been identified by a variety of methods and a growing number of specific lncRNAs have been demonstrated to affect genomic functions, including imprinting, enhancer function, X-chromosome inactivation, chromatin structure (including the lncRNA HOTAIR, which served as a scaffold to assemble and target Polycomb Raepressive Complex 2 and LSD1/CoREST/REST complexes to the HOXD locus and co-ordinated H3K27 methylation and H3K4 demethylation for affecting chromatin structure) and genomic rearrangements during the generation of antibody diversity (4). Multiple studies have demonstrated that significant numbers of lncRNAs are regulated during development, exhibit cell type-specific expression, localize to specific subcellular compartments and are associated with human diseases (1). Certain studies have revealed that lncRNAs are widely expressed in the mammalian nervous system and a large amount are likely to be important in neuronal development and activity (5,6). Furthermore, lncRNAs are now being implicated in neurodegenerative processes, including Huntington’s disease (HD), amyotrophic lateral sclerosis (ALS) and Alzheimer’s disease (AD) (5,6). Previous studies demonstrated that lncRNA caused increases in levels of taurine upregulated gene 1 and nuclear enriched abundant transcript 1 in the HD caudate, while maternally expressed 3 was downregulated (5). Furthermore, in ALS, fused in sarcoma/translocated in sarcoma (FUS/TLS) protein acts as an RNA binding protein that is able to be recruited by a lncRNA to the genomic locus encoding cyclin D1, where it represses cyclin D1 transcription. However, mutations in the *FUS*/*TLS* gene caused an lncRNA-mediated abnormality in cyclin D1 transcription regulation in a subset of ALS cases (5). In addition, the abnormal expression of certain lncRNAs, including ATXN8OS and the antisense transcript for β-secretase-1 (BACE1-AS) are closely correlated with AD. Certain observations suggested that the mutant lncRNA ATXN8OS transcript contributes to the pathogenesis of spinocerebellar ataxia type 8 by altering the activity of the MBNL/cellobiose-6-phosphate hydrolase alternative splicing protein in AD (5). By contrast, Faghihi *et al* identified a lncRNA conserved noncoding BACE1-AS that regulates the mRNA and protein expression of β-secretase-1 (BACE1) in the brain in an AD mouse model (7). Previous studies indicated that BACE1 is a crucial enzyme in AD pathophysiology (7,8). Sequential cleavage of amyloid precursor protein (APP) by the β-site cleaving enzyme BACE1, which is essential for amyloid β-protein (Aβ) 1–42 and Aβ_1–40_ biosynthesis, and secretase, initiates the ‘amyloid cascade’ that is central to the pathophysiology of AD (7,8). Furthermore, Aβ_1–42_ oligomers produced by BACE1 affect key aspects of AD (7–9). The results of the study by Faghihi *et al* demonstrated that lncRNA BACE1-AS is elevated in AD and drives the rapid feed-forward regulation of β-secretase (7). Although the functions of lncRNAs remain to be fully elucidated, lncRNA network changes in neurodegenerative processes may be important in understanding and treating the associated diseases. Based on previous evidence, the present study hypothesized that the inhibition of endogenous lncRNA BACE1-AS by RNAi silencing technology may attenuate the ability of BACE1 to cleave APP, thus delaying the production of Aβ_1–42_ oligomers. Therefore, the present study aimed to investigate this hypothesis in an *in vitro* senile plaque (SP) AD cell model using synthetic Aβ_1–42_-treated SH-SY5Y cells transfected with siRNA-BACE1-AS or siRNA-mock expression plasmid DNA.

## Materials and methods

### Cell culture and Aβ_1–42_ treatment

The AD SP cell model was generated as previously described (8). The SH-SY5Y cell lines were seeded in a six-well plate in Dulbecco’s modified Eagle’s medium supplemented with 10% fetal calf serum, penicillin (100 U/ml) and glutamine (0.3 mg/ml; all ingredients were purchased from Invitrogen Life Technologies, Grand Island, NY, USA) and incubated in a humidified tissue culture incubator containing 5% CO_2_ at 37°C until 80% confluence was achieved. Then, 10 μmol/l large aggregates of synthetic A_β1–42_ (Sigma-Aldrich, St. Louis, MO, USA) were added to the cultures. Following 24 h, the drug-containing medium was replaced with fresh normal cell medium for continued culture.

### MTT assay for cell proliferation

Each group of SH-SY5Y cells was seeded at 2×10^3^ cells per well in a 96-well plate until 85% confluent. MTT (Sigma-Aldrich) reagent (5 mg/ml) was added to the maintenance cell medium at different time-points and incubated at 37°C for an additional 4 h. The reaction was terminated with 150 μl dimethylsulfoxide (Sigma-Aldrich) per well, the cells were lysed for 15 min, and the plates were gently agitated for 5 min. The absorbance values were determined using an ELISA reader (Model 680; Bio-Rad, Hercules, CA, USA) at 490 nm.

### RNA extraction and analysis by quantitative polymerase chain reaction (qPCR)

Total RNA from each group was isolated with TRIzol reagent (Invitrogen Life Technologies), according to the manufacturer’s instructions. The RNA samples were treated with DNase I (Sigma-Aldrich), quantified, and reverse-transcribed into cDNA with the ReverTra Ace-α First Strand cDNA Synthesis kit [Toyobo (Shanghai) Biotech Co., Ltd., Shanghai, China]. qPCR was conducted using a RealPlex4 real-time PCR detection system from Eppendorf AG (Barkhausenweg, Hamburg, Germany), with SYBR-Green Real-time PCR Master mix [Toyobo (Shanghai) Biotech Co., Ltd.] as the detection dye. qPCR amplification was performed for >40 cycles with denaturation at 95°C for 15 sec and annealing at 57°C for 45 sec. Target cDNA was quantified with the Eppendorf BioSpectrometer (Eppendorf AG). A comparative threshold cycle (Ct) was used to determine gene expression relative to a control (calibrator), and steady-state mRNA levels are reported as an n-fold difference relative to the calibrator. For each sample, the marker gene Ct values were normalized using the following formula: ΔCt = Ct_genes − Ct_18S RNA. To determine relative expression levels, the following formula was used: ΔΔCt = ΔCt_samplegroups − ΔCt_controlgroup. The values used to plot the relative expression of the markers were calculated using the 2^−ΔΔCt^ method. The mRNA levels were calibrated on the basis of levels of 18S rRNA. The cDNA of each gene was amplified with primers as previously described (7). The following primers were used: BACE1, forward 5′-GCAGGGCTACTACGTGGAGA-3′ and reverse 5′-CAGCACCCACTGCAAAGTTA-3′; APP, forward 5′-TTTGGCACTGCTCCTGCT-3′ and reverse 5′-CCACAGAACATGGCAATCTG-3′; Ki67, forward 5′-TGGGTCTGTTATTGATGAGCC-3′ and reverse 5′-TGACTTCCTTCCATTCTGAAGAC-3′; 18s rRNA, forward 5′-CAGCCACCCGAGATTGAGCA-3′ and reverse 5′-TAGTAGCGACGGGCGGTGTG-3′.

### Western blot analysis

The cells were lysed using a 2X loading lysis buffer (Beyotime Institute of Biotechnology, Shanghai, China). The total amount of proteins from the cultured cells was subjected to 12% SDS-PAGE and transferred onto a hybrid polyvinylidene difluoride (PVDF) membrane (Millipore, Bedford, MA, USA). Following inhibition with 5% (w/v) non-fat dried milk in Tris-buffered saline with Tween-20 (TBST; Beyotime Institute of Biotechnology), the PVDF membranes were washed four times (15 min each) with TBST at room temperature and incubated with primary antibodies, including rabbit anti-human Ki67 antibody (Santa Cruz Biotechnology, Inc., Santa Cruz, CA, USA), rabbit anti-human BACE1, Aβ1–40, Aβ1–42 and GAPDH antibodies (Cell Signaling Technology, Inc., Beverly, MA, USA). Following extensive washing, the membranes were incubated with horseradish peroxidase (HRP)-conjugated goat anti-rabbit immunoglobulin (Ig) G secondary antibody (1:1,000; Santa Cruz Biotechnology, Inc.) for 1 h. Following washing four times (15 min each) with TBST at room temperature, the immunoreactivity was visualized using an enhanced chemiluminescence kit from Perkin Elmer, Inc. (Norwalk, CT, USA).

### Immunofluorescence (IF) staining

The cultured cells were washed three times with phosphate-buffered saline (PBS) and fixed with 4% paraformaldehyde (Sigma-Aldrich) for 30 min. Following inhibition, the cells were initially incubated with primary antibody overnight at 4°C, and then with fluorescein isothiocyanate- or Cy3-conjugated goat anti-rabbit IgG antibody (1:200; Sigma-Aldrich) and 5 μg/ml DAPI (Sigma-Aldrich) at room temperature for 30 min. Then, the cells were thoroughly washed with TBST and viewed through a fluorescence microscope (DMI3000; Leica, Allendale, NJ, USA).

### ELISA assay

The Aβ_1–42_ ELISA kit (Hermes Criterion Biotechnology, Vancouver, BC, Canada) was used according to the manufacturer’s instructions. Briefly, all the cells and supernatants were harvested and dissociated in 0.1 M Tris (pH 7.4) containing 1% Triton X-100 (Sigma-Aldrich) and 5 mM MgCl_2_ by sonication. The concentration of Aβ_1–42_ was measured and the data were normalized against the protein concentration and expressed as a nanogram of Aβ_1–42_ per milligram of total protein. All the samples were added to anti-Aβ_1–42_ antibody-precoated microtest wells and incubated for 60 min. Following washing three times, the HRP-conjugated detection antibodies were then added followed by the addition of the substrate solution. The absorbance was determined at a wavelength of 450 nm.

### RNA extraction and northern blot analysis

Northern blotting was performed as previously described (10–12). For all the groups, 20 μg of good quality total RNA was analyzed on a 7.5 M urea 12% polyacrylamide denaturing gel and transferred onto a Hybond N^+^ nylon membrane (Amersham, Freiburg, Germany). The membranes were crosslinked using ultraviolet light for 30 sec at 1,200 mjoule/cm^2^. Hybridization was performed with the antisense starfire probe to detect the lncRNA *BACE1*-*AS* fragments according to the manufacturer’s instructions (7). Following washing, the membranes were exposed for 20–40 h to Kodak XAR-5 films (Sigma-Aldrich). As a positive control, all the membranes were hybridized with a human U6 snRNA probe. The sequence was as follows: Human U6 snRNA, 5′-GCAGGGGCCATGCTAATCTTCTCTGTATCG-3′. The exposure times for the U6 control probe varied between 15 and 30 min.

### Ribonuclease protection assay (RPA)

As previously described (7), each RNA sample was treated with ribonuclease A+T (Sigma-Aldrich), which digests single stranded RNAs but not RNA duplexes. The RNA samples were incubated at 37°C for 60 min prior to treatment with an RNAse A+T cocktail (Sigma-Aldrich). Subsequently, the samples were incubated at 37°C for 30 min after addition of the RNAse cocktail, and treated with proteinase K. The RPA assay was then used to detect BACE1 and BACE1-AS employing two sets of probes by northern blotting. The first set of probes were designed to target the overlapping region of the BACE1 sense and antisense transcripts and the second set to target the non-overlapping region of these transcripts.

### siRNA and cell transfection

An siRNA targeted lncRNA BACE1-AS expression plasmid was constructed as previously described (7). SH-SY5Y cells were transfected with 0.3 μg siRNA-BACE1-AS or an siRNA-mock vector using Lipofectamine 2000 (Invitrogen Life Technologies), according to the manufacturer’s instructions.

### Statistical analysis

Each experiment was performed as least three times and the data are expressed as the mean ± standard error. The differences were evaluated using Student’s t-test. P<0.05 was considered to indicate a statistically significant difference. All statistical analyses were performed using the SPSS 10.0 statistical software package (SPSS, Inc., Chicago, IL, USA).

## Results

### Exogenous Aβ_1–42_ suppresses SH-SY5Y cell proliferation and induces AD relative protein expression

**Figure 1:**
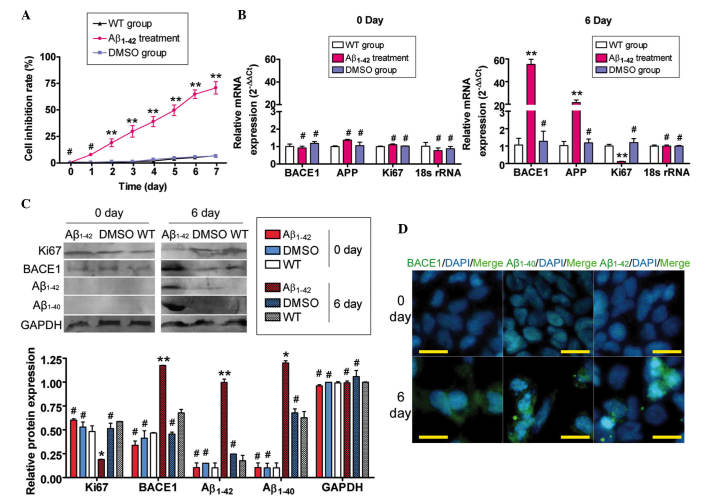
Exogenous Aβ1–42 affected SH-SY5Y cell proliferation and gene expression. (A) MTT assays demonstrated that large aggregates of synthetic Aβ1–42 inhibited SH-SY5Y cell proliferation in a time-dependent manner (^**^P<0.01 and ^#^P>0.05 vs. WT group; n=3). (B) Results of quantitative polymerase chain reaction analysis demonstrated that the mRNA expression of BACE1 and APP in the Aβ1–42 treatment group was markedly elevated, while the Ki67 expression in this group was markedly decreased compared with that in the other two groups on day six. However, no significant differences in the mRNA expression levels (normalized against 18S rRNA levels) of BACE1, APP and Ki67 were identified between the Aβ1–42-, the WT- and the DMSO- treated groups on day 0 (^**^P<0.01 and ^#^P>0.05 vs. WT group; n=3). (C) Western blot analysis confirmed that the expression of the BACE1, Aβ1–42 and Aβ1–40 proteins was significantly increased in the Aβ1–42 treatment group, compared with the WT- and DMSO-treated groups, while the expression of Ki67 in this group was markedly decreased on day six. GAPDH was used as a loading control (^**^P<0.01, ^*^P<0.05 and ^#^P>0.05 vs. WT group; n=3). (D) Immunofluorescent staining confirmed that the expression of the BACE1, Aβ1–42 and Aβ1–40 proteins was significantly increased in the Aβ1–42-treated group on day six, while the expression of these proteins was not detected on day zero (original magnification, ×200). Aβ, amyloid β-protein; WT, untreated group; APP, amyloid precursor protein; BACE1, β-secretase-1; DMSO, dimethylsulfoxide.

Firstly, the MTT assay was used to evaluate whether exogenous Aβ_1–42_ was able to suppress SH-SY5Y cell proliferation. Large aggregates of synthetic Aβ_1–42_ suppressed the proliferation of SH-SY5Y cells in a time-dependent manner (Fig. 1A). Next, the ability of exogenous Aβ_1–42_ to induce SP formation was assessed by qPCR, immunofluorescence (IF) staining and western blot analysis of the expression levels of APP-related factors. The qPCR results demonstrated that the expression of APP mRNA in the Aβ_1–42_-treated group was markedly elevated compared with that in the untreated (WT) and DMSO-treated control groups, while Ki67 expression was decreased on day six of Aβ_1–42_ treatment (Fig. 1). However, no significant difference in mRNA expression levels of APP and Ki67 was identified between the Aβ_1–42_-treated group and the control groups on day zero. Furthermore, western blot analysis confirmed that Aβ_1–42_ and Aβ_1–40_ protein expression was significantly increased in the Aβ_1–42_ treated group compared with the control groups, while Ki67 expression was markedly decreased on day six (Fig. 1). Additionally, IF staining confirmed the accumulation of Aβ_1–42_ and Aβ1–40 proteins, but not Ki67, in the Aβ_1–42_ treated group compared with the WT and DMSO-treated control groups on day six (Fig. 1). These data indicated that exogenous Aβ_1–42_ inhibited SH-SY5Y cell proliferation and induced the expression of APP-related factors and SP formation.

### Exogenous Aβ_1–42_ induces BACE1 and lncRNA BACE1-AS expression

**Figure 2:**
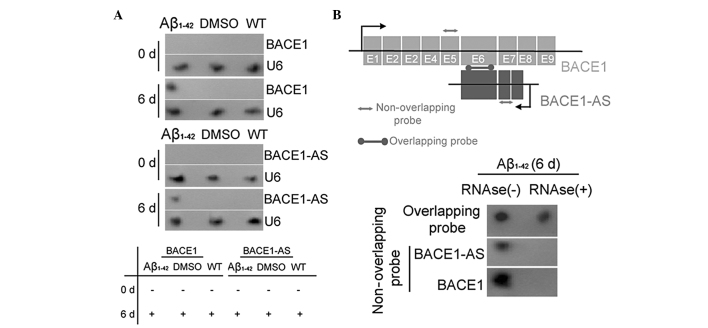
Exogenous Aβ1–42 induced the expression of the BACE1 enzyme and lncRNA BACE1-AS. (A) Northern blotting demonstrated that the BACE1 mRNA hybridization signals were higher in Aβ1–42-treated cell extracts than those in the other two groups. Strong lncRNA BACE1-AS hybridization signals were also detected only in Aβ1–42-treated cells. (B) RNA duplex formation. RNase protection assays were conducted on BACE1 mRNA to evaluate RNA duplex formation. Northern blot analysis revealed that non-overlapping probe hybridization signals were weaker than overlapping probe hybridization signals in SH-SY5Y cells following treatment with RNase, indicating that the overlapping part of BACE1 mRNA and lncRNA BACE1-AS transcripts was protected from degradation. These observations confirm that BACE1 and BACE1-AS form RNA duplexes. Aβ, amyloid β-protein; BACE1, β-secretase-1; lncRNA, long noncoding RNAs; BACE1-AS, antisense transcript for β-secretase-1; DMSO, dimethyl sulfoxide; WT, untreated group.

The expression of the enzyme BACE1 in SH-SY5Y cells, which is closely associated with Aβ_1–42_ processing, was investigated prior to (day zero) and following (day six) Aβ_1–42_ treatment using qPCR, IF staining and western blot analysis. The qPCR analysis demonstrated that the expression of BACE1 mRNA in the Aβ1–42-treated group was significantly elevated compared with that in the two control groups on day six (Fig. ). However, no significant differences in the mRNA expression levels of BACE1 in the three groups at day zero were identified. Western blotting confirmed that the BACE1 protein was expressed at significantly higher levels in the Aβ1–42 treated group than in the WT and DMSO-treated control groups on day six (Fig. 1). This pattern of expression was confirmed by IF staining of the BACE1 enzyme expression in SH-SY5Y cells on day six (Fig. 1). These data indicated that exogenous Aβ1–42 induced the expression of the APP-related processing enzyme BACE1. By contrast, northern blot analysis indicated that BACE1 mRNA and lncRNA BACE1-AS hybridization signals were higher in the extracts of Aβ1–42-treated cells than in those of the two control groups (Fig. 2A). RNase protection assays (RPA) were performed using BACE1 mRNA from each group to determine RNA duplex formation. Northern blotting revealed that non-overlapping probe hybridization signals were weaker than overlapping probe hybridization signals in SH-SY5Y cells following RNase treatment (Fig. 2B). This demonstrated that the overlapping part of BACE1 mRNA and lncRNA BACE1-AS transcripts were protected from degradation, thus, indicating that BACE1 and BACE1-AS indeed form an RNA duplex. These data indicated that exogenous Aβ1–42 not only promoted the expression of the APP-cleaving enzyme BACE1, but also induced lncRNA BACE1-AS expression. Furthermore, lncRNA BACE1-AS formed RNA duplexes with, and increased the stability of, BACE1 mRNA.

### Attenuation of the ability of BACE1 to cleave APP by siRNA silencing of lncRNA BACE1-AS expression

**Figure 3:**
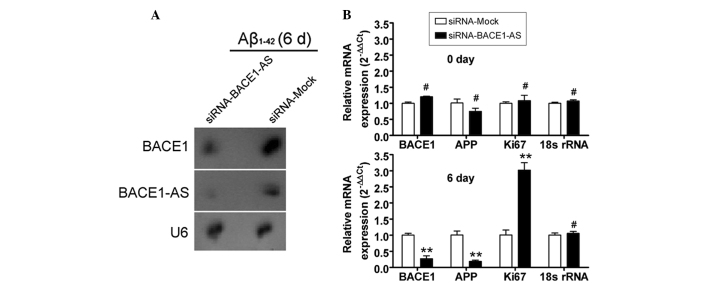
Attenuation of the ability of BACE1 to cleave APP by siRNA suppression of the expression of BACE1-AS. (A) Northern blotting demonstrated that BACE1-AS hybridization signals were weaker in siRNA-BACE1-AS-transfected cells than in siRNA-mock transfected cells. Strong BACE1 hybridization signals were detected in siRNA-mock transfected cells; however, not in siRNA-BACE1-AS transfected cells. (B) Quantitative polymerase chain reaction analysis demonstrated that the mRNA expression of APP and BACE1, but not that of Ki67, was significantly lower in siRNA-BACE1-AS-transfected cells than in siRNA mock-transfected cells on day six of Aβ1–42 treatment. However, no significant difference in the mRNA expression levels of APP, BACE1 and Ki67 (normalized against 18S rRNA expression) was identified in the two groups on day zero (^**^P<0.01 and ^#^P>0.05 vs. siRNA-mock transfected cells; n=3). APP, amyloid precursor protein; BACE1, β-secretase-1; BACE1-AS, antisense transcript for β-secretase-1; siRNA, small interfering RNA.

**Figure 4:**
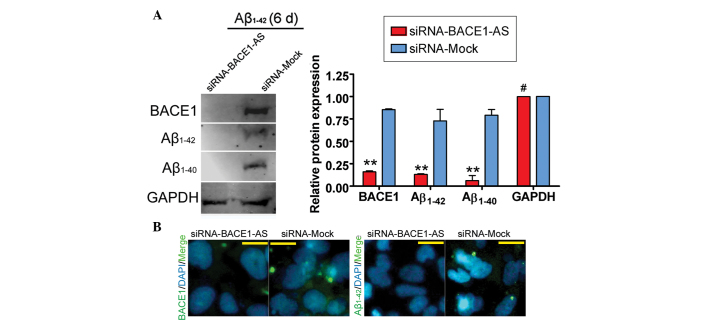
Inhibition of protein expression by siRNA suppression of BACE1-AS. (A) Western blot analysis revealed that the protein expression levels of BACE1, Aβ1–42 and Aβ1–40 were significantly decreased in siRNA-BACE1-AS transfected cells compared with siRNA-mock transfected cells. (^**^P<0.01 and ^#^P>0.05 vs. the siRNA-mock transfected cells group; n=3). GAPDH was used as a loading control. (B) Immunofluorescent staining confirmed that the expression of BACE1, Aβ1–42 and Aβ1–40 proteins was significantly decreased in siRNA-BACE1-AS-transfected cells compared with that in siRNA mock-transfected cells. BACE1, β-secretase-1; BACE1-AS, antisense transcript for β-secretase-1; Aβ, amyloid β-protein; siRNA, small interfering RNA.

The potential of siRNA silencing of lncRNA BACE1-AS expression to reduce the stability of BACE1 mRNA and to attenuate the ability of BACE1 to cleave APP was then investigated in SH-SY5Y cells. Northern blot analysis indicated that the lncRNA BACE1-AS hybridization signal was weaker in the siRNA-BACE1-AS-transfected cell group than that in the siRNA mock-transfected group (Fig. 3). Furthermore, a strong BACE1 hybridization signal was detected in the siRNA mock-transfected group, however, not in the siRNA-BACE1-AS-transfected group. In addition, qPCR analysis demonstrated that in siRNA-BACE1-AS-transfected cells, the expression levels of APP and BACE1 mRNA, however, not those of Ki67 mRNA, were significantly lower than those in the siRNA mock-transfected group on day six of Aβ1–42 treatment (Fig. 3). However, no significant differences in the expression levels of APP, BACE1 and Ki67 mRNA between the two groups were observed at day zero. Furthermore, IF staining and western blot analysis confirmed that the expression of BACE1, Aβ1–42 and Aβ1–40 proteins was significantly decreased in the siRNA-BACE1-AS-transfected group compared with that in the siRNA mock-transfected group (Fig. 4). These results indicated that the ability of BACE1 to cleave APP was decreased in SH-SY5Y cells by siRNA-mediated silencing of lncRNA BACE1-AS downregulation. These results also indicated that the stability of BACE1 in SH-SY5Y cells was closely associated with lncRNA BACE1-AS expression.

## Discussion

The nature and functions of ncRNAs appear to be numerous and varied. A range of small ncRNAs, including siRNAs, microRNAs and piRNAs, have been implicated in a host of roles, including transcriptional regulation, control of chromatin structure, heterochromatin formation and proteomic status (4). However, accumulating evidence indicated the existence in mammals of a specific class of ncRNA, namely lncRNAs, which vary in size from 200 bp to >1,000 bp, which is much larger than the variety of small ncRNAs that have been identified. Several studies have reported difficulty in cloning the full length of various lncRNAs, possibly due to the increased complexity in their structure compared with that of most small ncRNAs. By contrast, lncRNAs have a wide variety of sources and are involved in numerous processing and regulatory pathways. LncRNAs are transcribed by RNA polymerase II, and are often spliced and polyadenylated (4). They have been identified by a variety of methods and the number of specific lncRNAs demonstrated to affect genomic function is growing. These include lncRNAs with roles in imprinting, enhancer function, X chromosome inactivation, chromatin structure and genomic rearrangements during the generation of antibody diversity (4). Despite associations with a number of disorders, lncRNAs remain a relatively unexamined area in the study of diseases, and may represent a source of new therapeutic targets (1). To date, the majority of studies have indicated that lncRNAs act as negative regulators of their target genes. However, Faghihi *et al* (7) identified an lncRNA that acted as a positive regulator of its target gene in a study of the pathogenesis of AD (1,7). The study identified an lncRNA BACE1-AS gene, which generates amyloid β (Aβ). The lncRNA BACE1-AS increased the stability of the BACE1 mRNA, thus leading to the amplified production of Aβ peptides and the deleterious feed-forward cycles of disease progression (7). Based on these observations, the present study hypothesized that silencing the expression of endogenous lncRNA BACE1-AS diminishes Aβ formation and neuronal damage as a consequence. The present study demonstrated that β-secretase expression was significantly reduced at the mRNA and protein levels in SH-SY5Y cells as a result of siRNA-mediated silencing of lncRNA BACE1-AS expression. Furthermore, exogenous Aβ1–42 did not stimulate the formation of endogenous Aβ (1–40/1–42) in siRNA-BACE1-AS-transfected SH-SY5Y cells. These data indicated that the inhibition of the expression of lncRNA BACE1-AS effectively inhibited the endogenous production of Aβ peptides. By contrast, when the expression of lncRNA BACE1-AS was silenced in transfected SH-SY5Y cells, treatment with exogenous Aβ peptides had a significantly reduced cytotoxic effect and these cells maintained their normal state. Therefore, lncRNA BACE1-AS is likely to be an important factor in the formation of mature Aβ peptides. The ability of BACE1 to cleave APP was attenuated via silencing the expression of lncRNA BACE1-AS.
